# Molecular Pathology of Hepatic Neoplasms: Classification and Clinical Significance

**DOI:** 10.4061/2011/403929

**Published:** 2011-04-07

**Authors:** Zenta Walther, Dhanpat Jain

**Affiliations:** Department of Pathology, Yale University School of Medicine, 310 Cedar Street, P.O. Box 208023, New Haven, CT 06520-8023, USA

## Abstract

Recent technological advances have enabled investigators to characterize the molecular genetics and genomics of hepatic neoplasia in remarkable detail. From these studies, an increasing number of molecular markers are being identified that correlate with clinically important tumor phenotypes. This paper discusses current knowledge relevant to the molecular classification of epithelial primary hepatic tumors that arise in adults, including focal nodular hyperplasia (FNH), hepatocellular adenoma (HCA), hepatocellular carcinoma (HCC), cholangiocarcinoma (CC), and combined HCC-CC. Genetic analysis has defined molecular subtypes of HCA that are clinicopathologically distinct and can be distinguished through immunohistochemistry. Gene expression studies have identified molecular signatures of progression from dysplastic nodules (DNs) to early HCC in cirrhosis. Analyses of the mutational spectra, chromosomal aberrations and instability, transcriptomics, and microRNA profiles of HCC have revealed the existence of biologically distinct subtypes of this common malignancy, with prognostic implications. Molecular characterization of biliary and hepatic progenitor cell phenotypes in liver cancer has shed new light on the histogenesis of these tumors and has focused attention on novel therapeutic targets. In coming years, the molecular classification of hepatic neoplasms will be increasingly valuable for guiding patient care, as targeted therapies for liver cancer are developed and brought into clinical practice.

## 1. Introduction

Over the past decade, tremendous advances have been made in the technologies available for characterizing the molecular genetics, genomics and epigenetics of neoplasia. These advances have greatly accelerated basic research aimed at elucidating the molecular mechanisms underlying tumorigenesis. In addition, they have led to the identification of molecular markers that correlate with important biological characteristics of tumors. Such markers are increasingly valuable in clinical practice as tools for facilitating the diagnosis and categorization of tumors, determining their aggressiveness and, in some cases, predicting their responses to particular forms of therapy. The ideal classification system for any group of neoplasms would be based on our understanding of their ontogeny and would integrate histologic features with molecular data in a clinically meaningful way. In this paper, we will discuss the molecular biology of primary hepatic tumors, with particular emphasis on the roles of molecular characterization in clinical practice. We will focus on primary hepatic neoplasms that arise in adults, including hepatocellular adenoma (HCA), hepatocellular carcinoma (HCC), cholangiocarcinoma (CC), and the rare entity, combined hepatocellular and cholangiocarcinoma (HCC-CC). Focal nodular hyperplasia (FNH), although thought to be a nonneoplastic lesion, is often considered in the differential diagnosis of other hepatocytic tumors and, therefore, has also been included in this discussion. However, consideration of mesenchymal, hematopoietic and childhood liver tumors is beyond the scope of this review.

FNH is the most common of the benign hepatocellular tumors, arising in approximately 3% of adults [1, 2]. Although the other major type of benign hepatocellular tumor, HCA occurs much less frequently, with an estimated incidence in Europe and North America of 0.003% [3], its association with hemorrhage, rupture, and risk of malignant transformation necessitates its accurate diagnostic distinction from FNH. This is an area in which molecular characterization has become very informative and clinically useful. The most common primary hepatic malignancy is HCC, which constitutes 80–85% of all malignant epithelial neoplasms originating in the liver. HCC often arises in a background of cirrhosis and presents a global public health problem far greater than that of HCA. In the United States, the annual incidence of HCC has been rising over the past three decades [4]. The second most common primary hepatic malignancy is CC, constituting 15–20% of the total, and its incidence has also been rising. There has been an explosion of basic research in the field of primary hepatic epithelial cancers in recent years, and we are just beginning to understand their molecular pathogenesis. We anticipate rapid advancements over the next several years in personalized medical approaches to the treatment of liver cancer, as the molecular mechanisms of hepatic tumorigenesis are elucidated and molecular classification of these lesions is integrated into clinical study design [5].

## 2. Focal Nodular Hyperplasia

Focal nodular hyperplasia (FNH) is a common, benign hepatocellular lesion that is believed to arise in response to localized hyperperfusion of the liver parenchyma, typically in association with a microscopic arterial malformation [6, 7]. FNH frequently presents as an incidental hepatic mass noted in imaging studies, and it can often be diagnosed on the basis of its radiographic appearance alone. However, sometimes the diagnosis requires histologic confirmation. Although the gross morphology and microscopic appearance of FNH in resection specimens are usually characteristic and easily recognized, distinguishing FNH from HCA in needle biopsies can be difficult. The distinction is, however, of great importance because of the differing clinical management of these entities.

**Figure 1:**
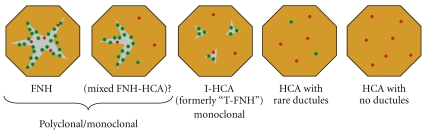
Schematic illustration of the histologic spectrum of benign hepatocellular proliferations. Polyclonal FNH is at one end of the spectrum and nontelangiectatic HCA is at the other; inflammatory HCA (I-HCA), formerly classified as telangiectatic FNH (T-FNH), has overlapping features and is shown in the middle. Gray areas indicate fibrosis; green and red dots represent bile ductules and arterioles, respectively.

Traditionally, FNH has been considered nonneoplastic and, accordingly, it is thought to have no malignant potential. However, molecular analyses of the clonality of FNH have yielded conflicting results, with some studies finding that up to 50% show X chromosome inactivation patterns suggestive of monoclonality [7, 8]. In general, polyclonality argues strongly against neoplasia, but the implications of monoclonality are less clear, since it may be influenced by factors such as variability in X-inactivation patterns, tissue architecture and turnover rate. Thus, the question of whether classic FNH is sometimes neoplastic remains controversial. Atypical forms of FNH have been described, including mixed lesions with both FNH-like and HCA-like areas, as well as lesions with histologic features intermediate between FNH and HCA [9]. The latter tumors were originally designated “telangiectatic FNH” (T-FNH), but subsequent molecular analyses have led to their reclassification as a variant of HCA (see below). Mixed FNH-HCA have not been subjected to molecular analysis and, indeed, have not been described in recent publications. Therefore, their existence as a distinct entity is uncertain. Although FNH and variants of HCA comprise a histologic spectrum (Figure 1), there is no compelling evidence that they are biologically related in the sense of one being the precursor of another.

Given the uncertainty surrounding the issue of neoplasia in FNH, several investigators have searched for genetic alterations that might define these lesions. If a genetic abnormality characteristic of FNH were found, it would greatly support its neoplastic nature, and the finding could be diagnostically useful in clinical practice. However, the results of such studies thus far have been uniformly negative, with no mutations detected in the APC, axin, *β*-catenin, HNF1*α*, or p53 genes in classic FNH [8, 10–12].

**Figure 2:**
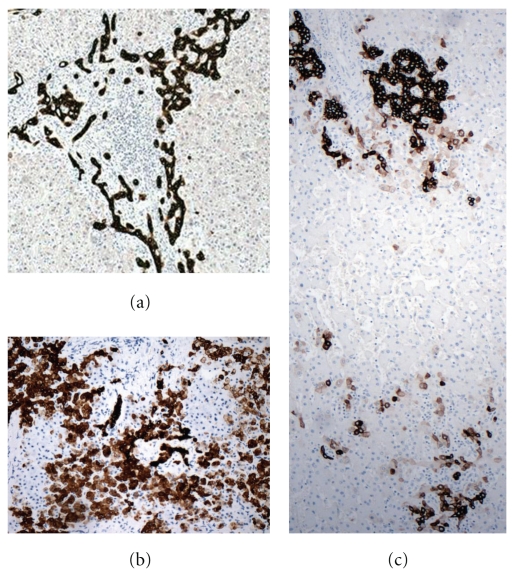
Patterns of immunohistochemical positivity for CK7 in benign hepatocellular lesions. (a) FNH displays strong CK7 staining of proliferating ductules but virtually no positivity within hepatocytes. (b) An example of HCA (nonmutated, noninflammatory type) showing focal, moderate CK7 staining within hepatocytes as well as heavy staining of a few bile ductules. (c) An I-HCA containing areas of bile ductular proliferation (arrow) with strong CK7 expression, resembling a ductular reaction in FNH, as well as other areas (e.g., bottom of image) in which there is focal hepatocellular staining for CK7, as is typical of HCA.

Both FNH and HCA consist predominantly of well-differentiated hepatocytes. In FNH, there are intervening fibrous bands that radiate from a central scar and contain abundant, proliferating bile ductules. In contrast, HCA have only rare bile ductules, if any, and typically show much less fibrosis. However, the regions of fibrous scar in FNH are variable in abundance and are not always adequately sampled in needle biopsies, sometimes making the histologic distinction from HCA difficult. We have found that the expression patterns of biliary markers, as determined by immunohistochemistry, are frequently different in FNH and HCA, not only within regions containing bile ductules, but also in hepatocellular areas [13]. Antibodies against cytokeratin 19 (CK19) and neuronal cell adhesion molecule (NCAM, also known as CD56), which are markers of hepatic progenitor cells as well as biliary epithelium, clearly stain the proliferating ductules within fibrous tracts in FNH and rare, isolated ductules in HCA. Cytokeratin 7 (CK7), which is known to be a marker of immature hepatocytes as well as progenitor cells and biliary epithelium [14], shows a distinct pattern of expression in HCA [15] that distinguishes the latter from FNH in most cases (Figure 2). In our experience, although occasional needle biopsies remain diagnostically challenging even after the combined use of immunohistochemical stains for CK7 and CK19, this analysis is usually quite helpful in the differential diagnosis of FNH and HCA [13, 16].

Immunohistochemical staining for the cytoplasmic enzyme, glutamine synthetase (GS), has also been proposed as a technique for differentiating between FNH and HCA [2]. GS is an enzyme that combines glutamate with ammonia to produce the amino acid glutamine. In normal liver, GS expression is limited to the centrilobular hepatocytes that directly border central veins, and its most important function is to aid in ammonia detoxification [17]. In recent years, the Wnt signaling pathway has been identified as a critical regulator of zonation in the liver [18]. Beta-catenin activation in perivenular hepatocytes, presumably the result of Wnt signaling from central veins, drives the expression of GS. In FNH, the centrilobular zones of *β*-catenin activation are expanded [12], and this leads to an irregular, geographic distribution of GS overexpression [2]. In HCA and HCC, several different patterns of GS expression have been observed, all distinct from that seen in FNH. As discussed later, activating mutations of the *β*-catenin gene occur in a subset of HCA. In adenomas with *β*-catenin mutation, GS is diffusely expressed, whereas in adenomas with normal *β*-catenin, GS is absent in large areas of the lesion. Unfortunately, however, in the latter type of HCA, irregular and/or centrizonal expression of GS can be present focally, particularly at the periphery of the tumor [2]. Therefore, although GS expression appears to robustly differentiate FNH from HCA in full resection specimens, it is less reliable for making this distinction in needle biopsies.

## 3. Hepatocellular Adenoma (HCA)

First described a half-century ago, HCA is a rare, benign liver tumor that most commonly occurs in women and has been associated with oral contraceptive use [19]. It is a neoplasm of demonstrated clonal origin [20, 21] and has a small but nonnegligible risk of malignant transformation [22]. Hemorrhage, pain, and rupture are other, more frequent complications of HCA, and their likelihood is proportional to the size of the tumor [1]. Histologically, HCA is a proliferation of mature-appearing hepatocytes arranged in cords one to two cells thick and lacking portal tracts. Isolated arterioles surrounded by hepatocytes and lacking a fibrous sheath (“naked” or “unpaired” arteries) can be seen scattered throughout the lesion. Arterioles accompanied by portal venules may be observed in a few portal-like structures, but these lack bile ducts. In some HCA, occasional isolated bile ductules may be seen, especially when immunohistochemical stains for CK7 or CK19 are applied.

In 2002, Bluteau et al. published the results of a genome-wide search for tumor suppressor genes in HCA [23]. Microsatellite analysis revealed a loss of heterozygosity (LOH) for markers at chromosome 12q caused by a small deletion of this region in five out of ten HCA. The TCF1 gene, encoding hepatocyte nuclear factor 1 (HNF1), was found to reside within the deletion. Because the function of HNF1 as a liver-specific transcription factor was already well established, this gene was pursued as the most likely candidate tumor suppressor affected by LOH in these adenomas. Further investigation confirmed that in ten out of sixteen HCA analyzed, HNF1 was somatically inactivated, either through a combination of gene deletion and mutation or via bi-allelic mutation. Interestingly, germline mutation of the HNF1 gene had been previously discovered to underlie a rare form of familial noninsulin dependent diabetes, MODY3 (maturity-onset diabetes of the young, type 3) [24]. This form of diabetes is associated with an increased risk of hepatic adenomatosis, due to random somatic inactivation of the second, nongermline-mutant HNF1 allele in the liver [25].

Shortly after genetic alterations of HNF1 in HCA were reported, a group in Taiwan published their discovery of activating mutations of the *β*-catenin gene in a fraction of these tumors. Earlier work by many independent groups had implicated Wnt pathway activation in liver carcinogenesis, and *β*-catenin mutations have been found in a significant percentage of HCC. To investigate the possibility that aberrant *β*-catenin signaling plays a role in very early hepatic neoplasia, Chen et al. performed directed LOH analysis and genomic sequencing of several Wnt pathway genes [10]. Three of ten HCA were found to bear small, in-frame deletions in one allele of the *β*-catenin gene, and to express truncated forms of the protein that are predicted to be constitutively activated.

The French group that had originally identified HNF1 inactivation in HCA next expanded their study to include assessment of *β*-catenin mutation status as well as genotype-phenotype correlations in these tumors [26]. In a collection of 96 HCA, 44 were found to have HNF1 gene inactivation and to display a characteristic histology, including marked hepatocellular steatosis, a lack of cytologic atypia, and an absence of inflammatory infiltrates in the lesion. Beta-catenin mutation was identified in 12 of the 96 tumors and was frequently associated with histologic features suggestive of malignancy, including nuclear atypia and pseudoacinar formation. Importantly, *β*-catenin mutation and HNF1 inactivation were mutually exclusive in these tumors, indicating the existence of at least two distinct, nonoverlapping molecular subtypes of HCA.

In the 1990s, a class of histologically unusual hepatic tumors was described and termed “telangiectatic FNH” (T-FNH) [9]. These lesions lacked a central scar, but because they displayed focal ductular reaction and a nodular architecture, they were categorized as an atypical variant of FNH. However, the sinusoidal dilatation, naked arterioles, and frequent intralesional hemorrhage that typified T-FNH were recognized as being similar to HCA (Figure 1). These peculiar features of T-FNH invited molecular characterization and comparison with both classic FNH and HCA. Two groups performed clonality assays and found a much higher rate of monoclonality in T-FNH than in classic FNH [11, 20]. The angiopoietin mRNA expression pattern, which had been shown to differ between FNH and HCA, was found in T-FNH to resemble that of HCA, and a global proteomic profile of T-FNH matched that of HCA while distinguishing both T-FNH and HCA from classic FNH [20]. Together, these findings strongly suggest that so-called telangiectatic FNH is actually a variant of HCA.

In the genotype-phenotype study described above, HCA that were found not to harbor genetic alterations in either HNF1 or *β*-catenin were further subdivided into two groups on the basis of the presence or absence of inflammatory infiltrates. The subgroup showing inflammation, named inflammatory HCA (I-HCA), was found to include all lesions formerly classified as T-FNH. Thus, four subtypes of HCA were proposed: (1) HNF1-inactivated HCA (H-HCA), (2) *β*-catenin-mutated HCA (*β*-HCA), (3) I-HCA, and (4) nonmutated, noninflammatory HCA [26]. A subsequent study of 93 HCA (47 of which had been previously analyzed) supported this classification system in general, except that *β*-catenin mutation was discovered to occur in a fraction of I-HCA as well as in noninflammatory HCA [27].

The hypothesis that I-HCA represents a biologically distinct entity was greatly strengthened with the finding of a marked overexpression of acute-phase reactants by the hepatocytes of these lesions [27]. At both the mRNA and the protein level, serum amyloid A (SAA) and C-reactive protein (CRP) are significantly overexpressed in I-HCA relative to nonneoplastic liver. When all types of hepatocellular adenoma are examined, the sensitivity and specificity of SAA overexpression by immunohistochemistry for identifying I-HCA was found to be 94%. The degree of SAA overexpression did not correlate with the extent of infiltration by inflammatory cells, which suggested that acute-phase protein expression might be an intrinsic feature of the neoplastic hepatocytes, whereas inflammatory infiltration might be secondary. To explore this possibility, Rebouissou et al. undertook a genome-wide mRNA expression study, comparing I-HCA to normal liver, and identified a pronounced activation of acute-phase inflammatory signaling in I-HCA [28]. Further inquiry into the potential causes of this inflammatory gene expression signature led to the discovery of mutations in the IL6ST gene, which encodes gp130, a component of the IL-6 receptor, in 60% of I-HCA [28]. These IL6ST mutations lead to ligand-independent activation of the IL-6 receptor, which promotes STAT3 signaling and induces the acute-phase inflammatory response within hepatocytes. The recruitment of inflammatory cells into I-HCA appears to be secondary to gp130-mediated hepatocellular production of the chemokine, CCL20, which attracts immune cells. In this study, all 43 I-HCA were found to show expression signatures indicative of IL-6 pathway activation, even those tumors in which no IL6ST mutation was identified. In I-HCA lacking IL6ST mutation, no other genetic alterations could be identified in various components of the STAT3 signaling pathway. However, gp130 protein levels were elevated in most of these lesions, suggesting that posttranslational control of gp130 was aberrant, perhaps due to occult mutation of a gene that normally regulates gp130's protein abundance.

**Figure 3:**
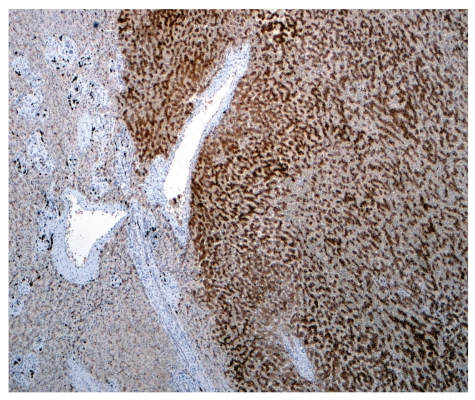
Immunohistochemical staining for serum amyloid A (SAA) in an inflammatory HCA shows strong, diffuse positivity in the tumor (right) but virtually no staining in adjacent normal liver (left).

In summary, HCA can be assigned to one of four categories [3]. The first is H-HCA and is defined by HNF1 inactivation. This variety accounts for 35–40% of all HCA and occurs almost exclusively in women. Rarely, these tumors arise in the context of familial diabetes (MODY3) and can be multiple. H-HCA can be identified through immunohistochemical staining for liver fatty acid binding protein (LFABP), which is a transcriptional target of HNF1 and is completely absent in the hepatocytes of these lesions. Histologically, H-HCA usually displays marked steatosis but no inflammation, and it lacks cytologic atypia. The second category, I-HCA, comprises over 50% of all HCA. It is characterized by the presence of inflammatory infiltrates, focal ductular reaction, sinusoidal dilation, and dystrophic arterioles. I-HCA almost invariably shows dramatic overexpression of acute-phase reactants, including SAA and CRP, which can be demonstrated by immunohistochemistry (Figure 3). Although the HNF1 gene is never inactivated in I-HCA, *β*-catenin mutations are sometimes found [29]. This type of HCA is associated with obesity, smoking, and alcohol consumption and is the most common variety of HCA in men. 

The third category of HCA is somewhat controversial and consists of lesions that harbor activating mutations of *β*-catenin but are noninflammatory; it has been reported that approximately 10% of adenomas belong to this *β*-HCA subtype. Beta-catenin activation is most easily assessed through immunostaining for glutamine synthetase, which is diffusely overexpressed in these lesions. Alternatively, immunohistochemical staining for *β*-catenin itself may be used to distinguish between tumors without mutation, in which *β*-catenin has a membranous localization, and those with mutation, in which an aberrant, activated form of *β*-catenin accumulates within nuclei. In both I-HCA and *β*-HCA, *β*-catenin mutation has been associated with a high risk of malignancy. These tumors are common in men, and they often show features, such as pseudoacini and hepatocellular dysplasia, that are frequent in HCC. In fact, because *β*-HCA may be histologically indistinguishable from well-differentiated HCC [28], their existence as a biologically distinct entity has been called into question. A group in the United States has recently reported their failure to find any examples of *β*-HCA in a collection of 41 adenomas subjected to molecular analysis [30]. We feel that in well-differentiated hepatocellular tumors, the presence of *β*-catenin mutation in association with morphologic features suspicious for malignancy should warrant a pathologic diagnosis of either outright or suspected hepatocellular carcinoma, rather than HCA. This is an area in need of further research to determine the molecular differences, if any exist, between *β*-HCA and HCC. Such findings would help to elucidate the pathogenesis of HCC and might serve as the basis for a diagnostic test of malignancy in histologically equivocal cases.

The fourth and final category of HCA are those that are noninflammatory (negative for acute phase markers) and do not harbor mutations in HNF1, *β*-catenin, or gp130. This is the smallest group of HCA, accounting for only about 5% of the total. Lesions that cannot be fully evaluated due to extensive necrosis and/or hemorrhage are also classified in this group. Whether these noninflammatory, nonmutant tumors represent a truly distinct subtype of HCA, or whether they are biologically related to one or more of the other three subtypes, is an open question that awaits further investigation.

## 4. Hepatocellular Carcinoma (HCC)

HCC almost always arises in the context of chronic liver disease, usually in patients with cirrhosis. Cirrhosis of any etiology constitutes the predominant risk factor for HCC. Worldwide, infection with hepatitis B virus (HBV) accounts for the majority of cases, whereas in the United States, chronic hepatitis C is the most common predisposing factor. HCC is often diagnosed late in the course of disease, but in developed countries, surveillance programs that use improved radiologic techniques for monitoring liver lesions in patients with cirrhosis are leading to increased rates of early HCC detection.

### 4.1. Dysplastic Nodules and Early Hepatocellular Carcinoma in Cirrhosis

It is now well accepted that in cirrhotic livers, the vast majority of HCC arise from benign precursor lesions called dysplastic nodules (DN) [31]. Although fully developed HCCs typically show histologic features that are easily recognizable to pathologists, the morphologic distinction between advanced DN and early HCCs is often more subtle and can be very difficult to identify, especially in needle biopsies [32]. As the molecular genetic events that drive the early steps of hepatic carcinogenesis are more fully elucidated, it is anticipated that molecular markers will be found that can help pathologists in making these clinically important distinctions. Indeed, in recent years, molecular analyses of the dysplasia-to-carcinoma pathway in cirrhosis have begun to yield valuable information for the recognition and classification of early hepatic neoplasia.

Several groups have used gene expression profiling to identify “molecular signatures” that can accurately distinguish between DN and early HCC [33–37]. Perhaps the most promising of these studies is that of Llovet et al., which analyzed the expression patterns of 55 hepatocarcinogenesis-related genes in dysplastic nodules and early HCCs of hepatitis C-infected patients. These authors identified a panel of only three genes (GPC3, LYVE1 and survivin) whose mRNA expression levels, as assessed by quantitative reverse transcription-PCR (qRT-PCR), were shown to correctly predict malignancy in 19 out of 20 early HCCs and nonmalignancy in 16 out of 17 DNs. As a test for HCC in this sample set, the sensitivity of this 3-gene expression panel was 95% and its specificity was 94%. Two of the genes in the panel, GPC3 (which encodes glypican-3) and survivin, are expressed by hepatocytes and are upregulated in HCCs, as compared to DN. The third gene, LYVE1, is expressed by endothelial cells and is downregulated in malignancy. All three genes had been previously implicated in hepatic carcinogenesis, and their differential expression levels in DNs and HCCs is most likely independent of the underlying cause of cirrhosis. The advantage of using qRT-PCR to identify this sort of molecular signature is that the method is highly quantitative, much more so than conventional immunohistochemistry, and, therefore, more objective. However, a disadvantage is that it must be performed on pure lesional tissue, which can be scarce in needle biopsies. Furthermore, the method requires the sacrifice of a small amount of lesional tissue for RNA extraction and thus necessitates the destruction of some potentially valuable histologic information. Nevertheless, in cases with an adequate amount of tissue, this qRT-PCR-based approach has the potential for considerable practical utility. If its predictive accuracy can be shown to extend to lesions that arise in cirrhosis of all etiologies, and if it can be successfully employed for the analysis of formalin-fixed needle biopsies, it may become a valuable adjunct to histology for differentiating between DN and early HCCs.

The gold standard for diagnosis of DN and early HCCs is still histology; in fact, internationally agreed upon histologic definitions of these lesions and a standardized nomenclature have only recently been formulated [32]. This morphologic classification system was developed and refined on the basis of meticulous histologic analyses of entire lesions within surgical resection specimens. It is recognized that needle biopsies of such lesions will often lack areas that are critical for making a histologic diagnosis of malignancy. In particular, the diagnosis of early HCCs may require identifying the invasion of lesional hepatocytes into portal tracts within the surrounding, benign parenchyma. Because biopsies frequently fail to sample such areas, molecular markers capable of distinguishing between dysplastic and malignant hepatocytes are urgently needed. However, because histology is still the gold standard for diagnosis, the immunohistochemical assessment of molecular markers, as an adjunct to standard H&E histology, is generally preferred over nucleic acid analysis in clinical practice.

Many immunohistochemical markers have been assessed individually, but what has emerged as most useful currently is a panel of three markers: GPC3, GS, and HSP-70. Glypican-3 was first identified as a gene whose mRNA is frequently expressed in HCC but not in benign, adult liver, HCA or CC [38]. Numerous laboratories have since confirmed the overexpression of GPC3 in HCC at the mRNA and protein levels. Its utility as an immunohistochemical marker for HCC was first demonstrated by Yamauchi et al., who found diffusely positive GPC3 staining of malignant hepatocytes in 84% of HCCs and only focal, weak staining in a small set of DN [39]. Similar results were obtained by Llovet et al. in the study cited above.

**Figure 4:**
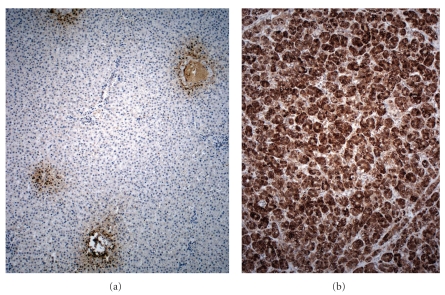
Glutamine synthase expression is (a) restricted to perivenular (zone 3) hepatocytes in normal liver but (b) diffuse and strong in HCC.

In an effort to augment the sensitivity of GPC3 immunostaining as a molecular test for HCC, and to retain its specificity in distinguishing DN from malignant lesions, Di Tommaso et al. examined the value of adding other tumor markers to the analysis [40]. Heat-shock protein 70 (HSP70) is an antiapoptotic, “stress response” gene that had been found through mRNA expression profiling to be markedly upregulated in early HCC, as compared with adjacent benign liver tissue [36]. Glutamine synthetase (GS) is a metabolic enzyme (now known to be upregulated by *β*-catenin signaling [41], as discussed above) that had also been shown to be overexpressed in primary liver cancer (Figure 4) [42]. When these three tumor markers, GPC3, HSP70, and GS, were applied as an immunohistochemical panel to a set of benign and malignant nodules that had been resected from cirrhotic livers, positivity for any two of the three markers was found to indicate malignancy with 72% sensitivity and 100% specificity. In a recent study, these authors used the same panel of immunostains to retrospectively analyze needle biopsies of a similar set of lesions and found that for distinguishing between high grade dysplastic nodules and well- or very well-differentiated HCC, the sensitivity of two-marker positivity as an indicator of malignancy decreased from 72% to 49%, while its specificity remained 100% [43]. This reduction in sensitivity may be attributable to sampling error and the somewhat heterogeneous expression patterns of HSP70 and GPC3 proteins within HCC. It would be interesting to know whether the mRNA expression patterns of GPC3, LYVE1, and survivin suffer from the same sort of intratumoral heterogeneity, which might complicate their use as molecular markers for HCC in needle biopsies.

Although immunohistochemical staining for the GPC3/HSP70/GS panel is currently recommended as the best ancillary technique to aid in the diagnosis of early HCC in difficult needle biopsy specimens [44], the search continues for biomarkers that can increase the sensitivity of this panel and that are easily and reproducibly stainable in tissue sections. Most candidate markers have been discovered through gene expression studies, and beyond their practical diagnostic utility, investigation into their functions in human liver and in model systems has yielded important insights into the mechanisms of hepatic carcinogenesis. A recent example of gene expression profiling leading to this kind of basic insight is provided by Kaposi-Novak et al. in their study of dysplastic nodules and early HCC in cirrhotic liver explants [37]. In addition to identifying molecular signatures able to discriminate between regenerative, dysplastic, and malignant nodules as well as confirming the upregulation of HSP70, GPC3, and several other putative tumor markers in early HCC, these investigators performed a comparative functional analysis of DN and HCCs gene expression signatures. Their results show that the gene expression profile of early HCCs differs from that of DNs in a pattern strongly suggestive of MYC oncogene activation. Interestingly, no mutations in the MYC gene were detected by sequencing of HCC genomic DNA, and MYC itself was not found to be overexpressed. However, a protein called CSN5 (also known as Jab1) that had been previously shown to posttranscriptionally promote MYC activation in breast epithelium [45] was found to be overexpressed in early HCC in parallel with induction of the MYC-regulated gene expression signature. This suggests a mechanistic model of early hepatocarcinogenesis in which an increase in the expression of CSN5 within DN leads to aberrant MYC activation, and thereby drives the progression of these lesions to HCC. If this model proves true, it may have profound implications for the molecular classification, diagnosis, and treatment of early HCC.

### 4.2. Hepatocellular Carcinoma Arising in Noncirrhotic Liver

The overall incidence of HCCs in patients without cirrhosis is difficult to estimate and varies with geography and the prevalence of risk factors in the population [46, 47]. Most cases occur in association with chronic liver disease, sometimes in a background of hepatic fibrosis that falls short of full-blown cirrhosis. Consequently, the risk factors for HCC development are the same in the absence of cirrhosis as in its presence; they include chronic HBV or HCV infection, chronic alcohol- or toxin-induced liver injury, and nonalcoholic steatohepatitis [46, 48]. Noncirrhotic patients with HCC are more likely than those with cirrhosis to have multiple risk factors. Although the molecular pathogenesis of hepatic malignancy is likely variable, with some genetic alterations more common in lesions that arise in association with particular risk factors than others, there is no strict correlation between the etiology of underlying chronic liver disease and histologic or molecular subtype of HCCs (see below). 

Hepatocellular carcinomas that arise in the absence of chronic liver disease or known risk factors for malignancy are almost always a histologic variant known as fibrolamellar HCC (FL-HCC). The great majority of these tumors occur in patients under the age of 35, and they display a characteristic histology, with nests of large, oncocytic, malignant hepatocytes surrounded by thick bands of layered fibrosis. Although the rarity of these tumors has hampered efforts to elucidate their molecular pathogenesis, FL-HCCs have been found to contain unique molecular alterations that distinguish them from the more common forms of HCCs (reviewed in [49]). The diagnosis of FL-HCCs depends mostly on histology, with the application of immunohistochemical markers when needed [50], and the recognition of clinical characteristics typical of this distinct clinicopathologic entity.

### 4.3. Hepatocellular Carcinoma Heterogeneity

Hepatocellular carcinoma has long been known to display extraordinary genetic complexity and molecular heterogeneity. However, it is not clear which of the many genomic, genetic, and epigenetic alterations found in HCCs are the most critical in driving its molecular evolution and/or defining its biological behavior. For this reason, there is currently no molecular subclassification of HCCs that is widely accepted and routinely implemented in clinical practice. Nonetheless, a host of molecular genetic markers have been found to correlate with clinical parameters, and in some instances, to have independent prognostic value in particular circumstances.

Although in recent years, surveillance of those at high risk of hepatic malignancy has initiated a trend toward earlier diagnosis of liver cancer, a substantial majority of HCC patients still present with locally advanced or metastatic disease [51]. Even among those whose cancer is diagnosed at a stage early enough to allow them to undergo potentially curative therapy (such as resection, percutaneous ablation, or liver transplantation), the rate of tumor recurrence remains significant. Overall long-term survival of patients with HCCs is poor, in part because most suffer from concomitant, underlying chronic liver disease. However, rates of recurrence and metastasis are not uniform, even within stage-matched patient groups, and there is evidence that from their beginnings, HCC tumors show individual variability in their degree of intrinsic biological aggressiveness [52]. In addition, the number of therapeutic options for HCCs is growing rapidly, due to advances in fields such as interventional radiology and transplantation as well as the emergence of a large armamentarium of molecularly targeted antineoplastic drugs [53, 54]. Thus, for HCC patients at all stages, there is a need for prognostic and predictive biomarkers that can aid in the subclassification of these tumors and help to identify patients who are most likely to respond to each available treatment modality.

### 4.4. Chromosomal Abnormalities and Genetic Mutations in HCC

HCCs generally show a high level of chromosomal instability, and this is a phenotype that is acquired early in the process of carcinogenesis [55]. Thus, chromosomal aberrations are common in this type of cancer. However, there is an enormous variety of alterations that can occur, and they have been found throughout the genome. Many of these chromosomal alterations are undoubtedly “passenger” changes, rather than the “drivers” of tumor progression. In the past decade, advances in genomics technologies such as the development of array-based comparative genomic hybridization (array CGH) have allowed investigators to map HCC-associated chromosomal alterations at a much higher resolution than was previously possible. A number of chromosomal regions have now been identified in which gains or losses occur frequently in HCCs (reviewed in [56]), and progress is being made in determining which genes are the targets of these recurrent changes. For example, by comparing array CGH data with global gene expression patterns (determined using DNA microarrays) in 49 HCC samples, Patil et al. were able to correlate the recurrent gain in chromosome 8q with Jab1 overexpression [57]. Interestingly, this is the same gene (also known as CSN5) whose overexpression was recently implicated in the activation of MYC that appears to drive the progression of DN to early HCC [37]. Thus, 8q gain may be a common mechanism promoting early hepatocarcinogenesis.

Although high-resolution mapping of chromosomal alterations in cancer is valuable mostly as a research tool, a group in Japan has demonstrated that HCCs can be subclassified into distinct, clinically meaningful groups based solely on their patterns of chromosomal alterations [58]. In this study, hierarchical cluster analysis was performed on the genomic gain/loss profiles of 87 HCCs, and the tumors were found to partition into two classes, termed A and B. Chromosomal alterations were more numerous in cluster A and typically included gains of 1q, 6p, and 8q as well as 8p losses; this group of tumors was associated with poor patient survival. Cluster A was further subdivided into three subgroups, each characterized by specific high-level amplifications (1q and 6p, 8q, and 17q). Cluster B contained two important subgroups, one without frequent chromosomal alterations and another with amplification of 17q. The authors reasoned that by analogy with known gene amplifications in other cancer types, the recurrent amplifications they discovered in HCC subgroups might indicate the presence of important oncogenes and that these oncogenes might be suitable therapeutic targets. Accordingly, the vascular endothelial growth factor A gene (VEGFA) was found to be contained within the amplified region of chromosome 6p in a subgroup of cluster A tumors, and a gene encoding an effector molecule of the mTOR signaling pathway was found to reside within the amplified region of 17q. The frequent high-level copy number gain of the 6p region encompassing VEGFA has been confirmed in a larger set of HCC tumors and correlated with VEGFA overexpression [59]. Both VEGF and the mTOR pathway are promising therapeutic targets in liver cancer. Therefore, the predictive value of amplifications at 6p and 17q in HCCs as molecular markers of response to VEGF- and mTOR-targeted therapies should be further explored in clinical studies.

The quantitative assessment of genomic alterations as an indicator of tumor aggressiveness has proven useful in predicting the risk of HCC recurrence after liver transplantation. In an effort to identify molecular markers of recurrence risk in HCC patients who had undergone transplantation, Marsh et al. analyzed explanted tumors for loss of heterozygosity (LOH) of microsatellite markers at nine tumor suppressor gene loci [60]. They found that the amount of allelic loss demonstrated within a particular tumor, when combined with other parameters such as tumor size and patient gender, correlated significantly with risk of tumor recurrence. In a subsequent study, the panel of microsatellite markers used in this analysis was refined, and a simple measure of the amount of LOH detected in each tumor was developed, called the fractional allelic imbalance (FAI) [61]. The measurement of FAI and determination of the presence or absence of macrovascular tumor invasion form the basis of the Pittsburgh staging system [62], which is reported to more accurately predict the risk of HCC recurrence after transplantation than staging systems based on radiologic and clinical parameters [63].

HCCs have been extensively analyzed for the detection of somatic mutations affecting known oncogenes and tumor suppressors (reviewed in [64]). The gene encoding *β*-catenin (CTNNB1), which is mutated in approximately 30% of HCC, is the oncogene most frequently activated in this cancer. HCC bearing *β*-catenin mutations are more likely than their nonmutant counterparts to show chromosome stability, an absence of HBV infection [65], a well-differentiated histology, and cholestasis [66]. However, there are conflicting data in the literature on the question of whether *β*-catenin mutation in HCC is associated with favorable or unfavorable prognosis [67, 68]. In general, tumor suppressor genes are more often mutated in HCC than oncogenes. The tumor suppressor most commonly mutated in HCC, overall, is TP53 (encoding the well-known cell cycle regulator, p53), but its frequency of mutation varies with geographical location. TP53 mutation in HCC occurs most commonly in Asia and Africa, where the combination of widespread dietary aflatoxin exposure and endemic hepatitis B fosters a high rate of mutagenesis in the liver [69]. In the West, TP53 mutations are considerably less frequent, affecting about 20% of HCCs. TP53 mutation in these tumors is associated with poor prognosis, chromosomal instability, and HBV infection [64].

### 4.5. Gene Expression Profiling and Molecular Subtypes of HCCs

A large number of studies have examined global gene expression patterns in HCCs using DNA microarray technology (reviewed in [70, 71]). The advantage of this approach is that it provides information about which signaling pathways and cellular processes are activated or suppressed in these tumors, regardless of the mechanisms (e.g., mutational or epigenetic) underlying the aberrant regulation. Indeed, transcriptomic analysis has led to the identification of reproducible HCC subtypes with differing cellular differentiation and biological behavior that correlate well with prognosis and may soon allow better patient stratification for treatment than current clinical HCC staging systems.

In 2004, a group led by Dr. Thorgeirsson at the National Cancer Institute published the results of a genome-wide expression study of 91 HCCs showing that the tumors fell into two subclasses with distinct mRNA expression profiles and dramatically different patient survival [72]. The poor survival group, designated A, displayed gene expression signatures of high cellular proliferation and low apoptotic rate. A subsequent study, in which human HCC expression profiles were compared with those of rodent hepatocytes (fetal hepatoblasts, adult hepatocytes, and genetically engineered murine HCC), led to further subdivision of group A HCCs into hepatoblast-like (HB) and mature hepatocyte-like (HC) subgroups [73]. The hepatoblast gene signature was associated with earlier recurrence and worse survival than the hepatocyte signature, and this association was independent of other pathologic variables. The authors speculated that the HB phenotype may reflect the origin of this subset of tumors from adult hepatic progenitor cells (HPCs), which, like fetal hepatoblasts, are able to differentiate into both cholangiocytes and hepatocytes. Consistent with this hypothesis, several well-known HPC markers such as cytokeratins 7 and 19 (CK7 and CK19) were found to be included in the HB gene signature.

The existence of two broad categories of HCCs, one with and the other without a “high-proliferation” gene expression signature, has been confirmed in multiple, independent studies [71]. Most have found that tumors of the high-proliferation group are more aggressive and less histologically differentiated than the group lacking this expression signature. Boyault et al. presented a remarkably detailed refinement of this molecular classification system, in which three subgroups of each major category of HCC were identified (yielding a total of six HCC subtypes) through expression analysis followed by correlation with various genetic, genomic, and clinical factors [74]. Although not yet clinically applicable, this sort of analysis helps to elucidate the biological basis of HCC heterogeneity and provides a foundation for future prospective studies aimed at correlating treatment responses with molecular subtype classification.

The epithelial cell adhesion molecule, EpCAM, is a known marker of fetal hepatoblasts and adult hepatic progenitor cells as well as biliary epithelium. In 2004, Kim et al. performed microarray studies of cirrhotic liver and found that EpCAM is dramatically overexpressed in premalignant lesions as well as in a subset of HCC [75]. Molecular characterization of the EpCAM-expressing HCC subgroup demonstrated a signature of coexpressed genes that included other HPC markers, such as c-kit and CK19 [76]. The further stratification of tumors into four groups according to EpCAM expression and the patients' *α*-fetoprotein (AFP) status (positive or negative for elevated serum AFP) was shown to define four prognostic categories, each with a characteristic gene expression pattern. Importantly, this result was replicated in three independent cohorts of HCC (all HBV-related), in one of which the EpCAM analysis was performed by immunohistochemistry, rather than mRNA hybridization. Thus, the EpCAM-AFP classification system has the potential for easy application in clinical practice. It is not yet clear whether the stratification of AFP-positive cases according to EpCAM expression will be clinically useful as a prognostic indicator, since AFP positivity is already well-known to be associated with aggressiveness in HCC [77]. However, there is evidence that EpCAM expression correlates with biological variables that can be targeted therapeutically, and, therefore, it may prove valuable as a predictive marker of responsiveness to antiangiogenic therapies [78], Wnt/*β*-catenin pathway inhibitors, and/or stem-cell targeted agents such as anti-EpCAM antibodies [79].

**Figure 5:**
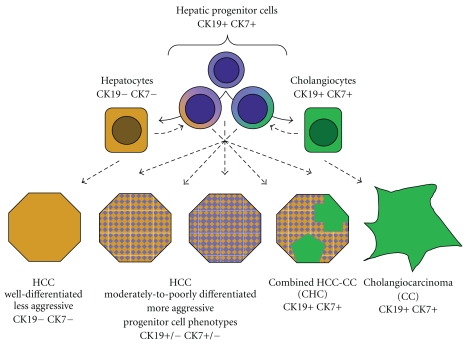
Hypothetical classification of primary hepatic malignancies according to corresponding patterns of hepatic progenitor cell differentiation. Dashed lines illustrate possible lineages of tumor origin and evolution. At left is well-differentiated HCC, with a mature hepatocytic phenotype (orange), and at right is cholangiocarcinoma, showing complete biliary differentiation (green). Varying degrees and patterns of expression of progenitor cell markers (purple) and biliary-type cytokeratins (CK7, CK19) correlate with increased tumor aggressiveness.

Gene expression profiling has yielded novel insights into the biological heterogeneity of HCCs and has particularly highlighted the importance of hepatic progenitor cell/stem-cell characteristics in defining its aggressiveness. In addition, these studies have focused attention on the roles of “cancer stem-cells” in HCCs [79]. However, much remains to be learned about the origins and behavior of hepatic progenitor cell lineages in benign and premalignant conditions, as well as their relationships to cancer stem-cells, cell-population dynamics within tumors, and the clinicopathologic ramifications of stem-cell marker expression in different HCC molecular subtypes (Figure 5). Although it is not yet clear which gene signatures and molecular markers will have the broadest applicability, recent rapid progress in this field implies that the incorporation of molecular data into HCC classification and staging systems in the near future will lead to continued improvements in the clinical management of HCC.

### 4.6. MicroRNA Profiling of HCCs

MicroRNAs (miRNAs) are short, noncoding RNA molecules that regulate gene expression by binding to specific messenger RNAs and preventing their translation into protein. Because each type of miRNA is able to downregulate hundreds of genes at a time, miRNAs often control entire transcriptional programs that determine fundamental cellular properties and behavior. Accordingly, miRNA profiling has emerged as an extremely valuable method for phenotyping and subclassifying tumors [80]. Compared to conventional gene expression profiling (in which protein-coding, messenger RNAs are examined), miRNA analysis has several advantages. Due to the stability of miRNAs, formalin-fixed samples (rather than frozen tissue) can be used. Furthermore, the interrogation of hundreds of miRNAs (and often significantly fewer) yields as much information as might be gleaned from examining thousands of messenger RNAs.

Many independent groups have conducted comprehensive analyses of miRNAs in HCC, and a plethora of informative miRNA markers have been identified. Many of these miRNA signatures correlate with important biological parameters, such as metastasis [81–83], differentiation [83–85], HBV or HCV infection [86, 87], tumor recurrence [88], and patient survival [89–91]. In addition to providing new candidates for investigation as possible diagnostic, prognostic, and/or predictive molecular markers in HCCs, these studies are opening new avenues in basic research on the mechanisms of hepatocarcinogenesis. For example, Ji et al. [92] have discovered that miRNA-181 is overexpressed in EpCAM-positive HCC cells and is a critical, functional determinant of the progenitor phenotype in these cells. MiRNA-181 appears to promote a HPC phenotype by downregulating the expression of key transcription factors that mediate the differentiation and maturation of hepatocytes.

It has been shown recently that the reduced expression (silencing) of miRNA-26 in HCC tumors of male patients is associated both with poor survival and response to interferon-alpha adjuvant therapy [89]. Interestingly, miRNA-26 is differentially expressed according to gender in benign liver, with significantly higher expression in women than in men. Among individuals with HCC, those whose benign liver tissue showed low miRNA-26 expression were found to have tumors in which miRNA-26 was even further downregulated, whereas in patients whose benign liver miRNA-26 expression levels were high, tumor levels of this miRNA were unchanged. These findings suggested that miRNA-26 acts as a tumor suppressor in the liver and that its higher expression in females may be a protective factor against HCC and may contribute to the marked gender bias in risk for this malignancy. Intriguingly, an analysis of gene expression in tumors that had undergone miRNA-26 silencing revealed the activation of interleukin-6 (IL-6) signaling networks. This is interesting in light of recent evidence that estrogen-related inhibition of IL-6 signaling underlies the observed female-specific resistance to hepatocarcinogenesis in mice [93]. An even more exciting finding with regard to miRNA-26 in human HCC was that miRNA-26 silencing within tumors identified the subgroup of patients who benefited from postoperative treatment with interferon-alpha after HCC resection in randomized trials [89]. The idea that the tumors most likely to respond to interferon treatment are those in which miRNA-26 silencing has led to increased proinflammatory and IL-6 signaling is intuitively appealing. Future prospective studies will undoubtedly test the efficacy of interferon-alpha in preventing recurrence of miRNA-26-silenced HCC in broader patient populations, as well as its value in combination with other therapeutic regimens. Thus, miRNA-26 promises to become the first predictive molecular marker in HCC that can be used to match individual patients with the treatments most likely to benefit them.

## 5. Intrahepatic Cholangiocarcinoma and Combined HCC-CC

Cholangiocarcinoma (CC), defined as carcinoma of biliary-type epithelium, can arise in the liver parenchyma or anywhere along the extrahepatic biliary tract. The extrahepatic type (which includes “Klatskin tumors” of the liver hilum) is much more frequent, accounting for up to 90% of CC cases [94]. The histologic appearance of intrahepatic CC (ICC) is the same as that of extrahepatic forms, typically showing malignant glands embedded in a prominent desmoplastic stroma. Although these morphologic features usually make ICC clearly distinguishable from HCC (a distinction that can be confirmed through staining for the hepatocytic marker, HepPar-1, and the biliary markers, CK7 and CK19), it can be difficult to differentiate between ICC and metastasis to the liver from a nonhepatic primary tumor. A few new molecular markers of biliary differentiation have recently been described [95], but these have not yet been tested for their ability to distinguish ICC from hepatic metastasis.

CC generally presents at a late stage, when curative resection is no longer possible. Unfortunately, this malignancy is highly aggressive and resistant to standard chemotherapeutic regimens. Molecular characterization of CC is in the early investigative stages (reviewed in [96]), and useful prognostic molecular markers have not yet been reported. The need for more research in this area, and for the development of targeted therapies and predictive molecular markers, is well recognized.

Combined HCC-CC is a rare primary liver tumor characterized by the intimate intermingling of histologically unequivocal HCC and CC components [97]. Most cases show, in addition to typical HCC and CC areas, transitional regions containing immature-appearing cells that express hepatic progenitor markers such as EpCAM. When such transitional regions predominate, the tumor is classified as a subtype of HCC-CC, “with stem-cell features” [98]. Three histologic patterns of intermediate/stem-cell distribution have been described in lesions of this category, including a pattern sometimes termed cholangiolocarcinoma, formerly classified as a variant of CC. Although several immunohistochemical studies of HCC-CC have confirmed the expression of various hepatic progenitor cell markers in these lesions [99, 100], their classification is based on histomorphology, rather than positivity for stem-cell markers *per se*. The fact that many HPC markers are also expressed by biliary epithelium and the recent findings on progenitor cell marker expression by HCC greatly complicate this field. Thus, although there is evidence that HCC-CC arises from a bipotential hepatic progenitor cell, the histogenesis of this rare neoplasm is still debated (Figure 5).

Only a few studies have examined the molecular characteristics of HCC-CC. Zucman-Rossi's group in France used microsatellite-based LOH analysis to assess chromosome stability in 9 CC, 15 HCC-CC, and 3 HCC/CC collision tumors [101]. Combined HCC-CC was found to show a high degree of chromosomal instability, similar to CC and unlike HCC. Investigators in Germany recently reported the results of CGH analysis of 49 HCC, 22 hepatic CC, and 7 HCC-CC cases [102]. In this study, combined HCC-CC was found to resemble both HCC and CC, showing several of the specific chromosomal gains and losses typical of HCC, while also displaying a high total number of chromosomal imbalances, similar to CC. Woo et al. recently performed gene expression profiling of 70 HCC, 13 CC, and 7 HCC-CC [103]. Results showed that the gene expression pattern of HCC was markedly different from that of CC, and combined HCC-CC clearly clustered with CC. Further analysis led to the identification of a CC gene signature containing many known biliary and hepatic progenitor cell markers. Interestingly, closer examination of the HCC data revealed that a subset of HCC also expressed the CC gene signature; this subset was designated CC-like HCC (CLHCC). Expression of the CC gene signature by HCCs was associated with poor prognosis. However, its prognostic significance was found to be independent of previously described poor-prognostic gene signatures such as that of the hepatoblast subtype of HCC. Thus, there are many layers of complexity regarding hepatic progenitor cell and biliary phenotypes in primary liver cancers. Overall, stem-cell and biliary characteristics seem to correlate with increased tumor aggressiveness, resistance to chemotherapy, and poor prognosis.

## 6. Conclusions

In recent years, remarkable progress has been made in elucidating the molecular pathology of hepatic tumors. In the area of benign hepatocellular lesions, advances in our understanding of molecular subtypes have led to a new classification system of HCA, with important genotype-phenotype correlations and easy application to routine clinical practice. In the field of early hepatic neoplasia, microarray studies of gene expression have identified simple molecular signatures that can distinguish dysplastic nodules from early HCCs in a background of cirrhosis. Although such expression signatures can be determined by quantitative RT-PCR, the use of immunohistochemistry, which allows the simultaneous assessment of histology and protein expression, is preferred in needle biopsies. Molecular measures of prognosis in patients with HCCs and CC are emerging; in retrospective studies, a variety of molecular techniques have successfully identified subgroups of HCCs with distinct clinical associations, such as prognosis, risk of recurrence after attempted curative treatment, and response to adjuvant interferon therapy. Although these assays are not yet developed for widespread clinical application, their refinement and use for the stratification of patients undergoing treatment in clinical trials will lead to further advances in therapeutic options for liver cancer. The current system of classifying hepatic tumors based on histologic architecture and patterns of differentiation has many limitations. Molecular studies have supported a model of hepatic tumorigenesis in which neoplasms recapitulate various lineages and stages of hepatic progenitor cell differentiation. These discoveries are likely to have an important impact on liver tumor classification and to lead to a newer classification system, based on histologic and molecular features, with improved prognostic and therapeutic significance.
